# Restricted visual scanpaths and hyperarousal during emotion recognition in childhood social anxiety disorder

**DOI:** 10.1192/j.eurpsy.2021.251

**Published:** 2021-08-13

**Authors:** J. Högström, J. Lundin Kleberg, E. Serlachius

**Affiliations:** Clinical Neuroscience, Karolinska Institutet, Stockholm, Sweden

**Keywords:** social anxiety disorder, eye tracking, scanpaths, Children and Adolescents

## Abstract

**Introduction:**

Social anxiety disorder (SAD) typically develops during late childhood or early adolescence, and often runs a chronic course if left untreated. Maladaptive processing of social information has been suggested to contribute to the etiology and maintenance of SAD. Scanpaths are a succession of visual fixations and saccades through which individuals extract information during face perception. Atypically long scanpaths have previously been reported in adults with SAD but no studies have been conducted on youth samples. SAD has previously also been linked to atypical arousal during face processing.

**Objectives:**

This study aimed to investigate differences in visual attention and arousal to emotional faces comparing children and adolescents with SAD to a non-psychiatric population of youths.

**Methods:**

In one of the largest eye-tracking studies of pediatric SAD to date, children and adolescents with SAD (n = 62) and healthy controls (n = 39) completed a task where they were meant to recognise different emotional expressions in pictures of faces while their eye movements were recorded. The visual scanpath and the pupil dilation response were examined.

**Results:**

Youth with SAD showed restricted scanpaths, suggesting they scanned a more limited part of the face during face perception. Higher pupil dilation was also observed in the children and adolescents with SAD.

**Conclusions:**

The restricted pattern of scanpath observed in youth with SAD is contrary to findings among adults, but similar to what has been reported in neurodevelopmental disorders associated with social interaction impairments such as autism. Restricted scanpaths may partially contribute to the maintencance of social anxiety disorder.

**Disclosure:**

No significant relationships.

